# The perspectives of patients with lithium-induced end-stage renal disease

**DOI:** 10.1186/s40345-018-0121-0

**Published:** 2018-06-02

**Authors:** Angèle P. M. Kerckhoffs, Erwin G. T. M. Hartong, Koen P. Grootens

**Affiliations:** 1 Jeroen Bosch Hospital, ‘s-Hertogenbosch, The Netherlands; 2 CWZ Hospital, Nijmegen, The Netherlands; 3 Reinier van Arkel, ‘s-Hertogenbosch, The Netherlands; 4 Radboudumc, Nijmegen, The Netherlands

## Abstract

**Background:**

Lithium is the treatment of choice for patients suffering from bipolar disorder (BD) but prolonged use induces renal dysfunction in at least 20% of patient. Intensive monitoring of kidney functioning helps to reveal early decline in renal failure. This study investigates the views and experiences of BD patients who have developed end-stage renal disease and were receiving renal replacement therapy.

**Results:**

The patients overall reported not to have been offered alternative treatment options at the start of lithium therapy or when renal functions deteriorated. All indicated to have lacked sound information and dialogue in accordance with shared decision making. Kidney monitoring was inadequate in many cases and decision making rushed.

**Conclusions:**

Retrospectively, the treatment and monitoring of lithium and the information process were inadequate in many cases. We give suggestions on how to inform patients taking lithium for their BD timely and adequately on the course of renal function loss in the various stages of their treatment.

## Background

As it can be very effective in the acute phase and maintenance of bipolar disorder (BD), and in the prevention of suicide, lithium therapy (LT) is considered the treatment of choice for BD (Goodwin et al. 2016; Miura et al. 2014; Cipriani et al. 2005). Lithium exposure is associated with an increased diagnostic incidence of moderate renal impairment. About 20% of patients on prolonged lithium therapy (LT) develop chronic kidney disease (CKD) (Bendz et al. 2010), Nielsen et al. recently reviewed the data on development of end-stage renal disease (ESRD) and showed they are diverse, from a rate of 5.3/1000 in older studies compared to no difference in prevalence in more recent studies (Nielsen et al. 2018). The improved renal outcome in the recent studies might be due to currently improved renal monitoring and a better focus serum lithium levels in patients with renal failure.

The renal complications warrant monitoring of kidney functions. International nephrology guidelines recommend discontinuation of LT in patients with a GFR < 60 ml/min per 1.73 m^2^, with the majority showing improvement or stabilisation of renal function when lithium therapy is discontinued at a renal clearance of 40 ml/min (Presne et al. 2003; Lepkifker et al. 2004). But what to do when clearance of 40 ml/min or lower or progressive loss of 5 ml/min per year indicates LT cessation while such an interruption carries a high risk of recurrence of the bipolar disorder. What to recommend patients facing the dilemma of progression to ESRD and renal replacement therapy (RRT) or acute episodes?

### Patient-centred care

Clinicians, patients, and others involved in their care and personal life typically base their treatment choices on scientific knowledge, clinical experience, and the patient’s values and preferences (Oxman et al. 2001). Due to its onset in early adulthood, its episodic course, the patient’s and family’s accumulating experience with and insight into individual manifestations and disease course, patient involvement tends to be high in BD, with patients exhibiting a high sense of responsibility for treatment decisions. Although the patient’s perspective hence deserves a leading role in the treatment of this potentially devastating disorder, studies reporting patient experiences with BD and LT are rare (Fisher et al. 2017).

### LT and ESRD from the patients’ perspective

Most patients and clinicians are aware of the effectiveness of LT on quality of life but also of its longer-term risks. A minority of patients will develop CKD and some even ESRD with prolonged use, rendering active monitoring for signs of renal involvement indispensable. In this study we focus on the experiences of patients with LT-induced ESRD receiving RRT. What are their views on the consequences of their often prolonged LT? Do their reports on treatment choices reflect shared decision making? Did they receive sound information on the pros and cons of LT to base their decisions on? Did they know of the risks of CKD, ESRD, and RRT and what was their attitude towards these potential adverse effects? Were they actively involved in the choice of treatment strategies? Were they ever faced with the dilemma of stopping successful LT at the cost of a raised recurrence risk? We are unaware of any study of ESRD patients reporting on these aspects. In this study we posed these important questions to ten such patients.

## Methods

### Patients

The Dutch register of RRT patients was consulted for BD patients with LT-induced ESRD. Ten patients were subsequently interviewed of whom informed consent was obtained to audiotape the interviews for later analysis and to use anonymous quotations.

### Interviews

The first author (AK) gauged the patients’ experiences during a semi-structured interview lasting a mean of 51 min. The following topics were addressed: (1) information received about the diagnosis and LT, (2) information received about adverse effects of LT, (3) experiences with RRT, and (4) suggestions for BD patients facing similar dilemmas. The content of the interview was jointly determined by the authors in two consensus meetings. All authors are clinicians with experience with the topic and working in lithium treatment units specialised in the care for CKD and ESRD patients (KG, EH) or delivering RRT (AK). After the third interview, we re-evaluated the content, without making any modifications except for change in the order of the questions.

### Quality analysis

The interview recordings were coded for thematic labels by AK and subsequently validated by KG and EH. The patients were asked to validate their answers to avoid misinterpretation. The results are presented in the order in which the themes were mentioned above.

## Results

### Patients

Ten patients diagnosed with BP and LT-induced ESRD receiving RRT were invited by their nephrologists to take part in the present study; all ten agreed to participate. The participants’ mean age was 71 years (range: 61–88 years) with nine patients being female. The sample was diverse. Eight patients were receiving outpatient haemodialysis, one patient was scheduled to start haemodialysis shortly, and one patient was receiving peritoneal dialysis. One of the patients was shortly to receive a kidney transplant while another was scheduled for home haemodialysis. Three patients were residents of assisted living facilities. Table 1 lists the answers per patient for three decision-making stages: at the start of LT, at the first signs of renal insufficiency and when renal failure was diagnosed. The table also shows the patients’ insight into their mental illness or psychiatric symptoms and their experiences with renal function monitoring.

**Table 1:** Interview results per patient

	No patients	Patient 1	Patient 2	Patient 3	Patient 4	Patient 5	Patient 6	Patient 7	Patient 8	Patient 9	Patient 10
Stage 1: start of lithium therapy (LT)											
Is patient aware of his/her BD diagnosis?	9/10	Yes	Yes	Yes	Yes	Yes	Yes	Yes	No	Yes	Yes
Did psych explain diagnosis?	6/10	No	No	Yes	Yes	Yes	Yes	Yes	No	Yes	No
Did psych discuss treatment options?	0/10	No	No	No	No	No	No	No	No	No	No
Could patient and/or family indicate own views/standards relevant for treatment decision?	1/10	No	No	No	No	No	Yes	No	No	No	No
Who took treatment decision?	10/10	Psych	Psych	Psych	Psych	Psych	Psych	Psych	Psych	Psych	Psych
Satisfied with decision-making process?	3/10	No	No	No	No	Yes	Yes	No	No	Yes	No
Stage 2: first signs of renal dysfunction/insufficiency											
Did psych/GP explain clinical problem?	4/6	No	n.a.	No	n.a.	Yes	n.a.	Yes	Yes	Yes	n.a.
Was neph involved at this stage?	4/6	Yes	No	?	No	Yes	No	Yes	No	Yes	No
Did psych/GP discuss alternatives to LT?	0/6	No	n.a.	No	n.a.	No	n.a.	No	No	No	n.a.
Could own views/standards be given for consideration in treatment decision?	1/6	No	n.a.	No	n.a.	No	n.a.	No	No	Yes	n.a.
Who took treatment decision? Psych/neph/patient/shared decision		neph	n.a.	Psych	n.a.	Psych	n.a.	Shared	Psych	Shared	n.a.
Satisfied with decision-making process?	4/6	Yes	n.a.	?	n.a.	No	n.a.	Yes	No	Yes	n.a.
Stage 3: renal failure											
Did psych/GP/neph explain situation?	8/10	Yes	Yes	Yes	Yes	Yes	No	Yes	Yes	Yes	No
Did psych/GP discuss alternatives to LT/?	2/10	No	Yes	No	No	Yes	No	No	No	No	No
Could own views/standards be given for consideration in treatment decision?	4/10	No	No	Yes	No	Yes	No	Yes	No	Yes	No
Who took treatment decision? Psych/neph/patient/shared decision		Psych	Psych	Shared	Psych	Shared	Psych	Shared	Psych	Shared	neph
Satisfied with decision-making process?	5/10	Yes	No	Yes	No	Yes	No	Yes	No	Yes	No
Psychiatric items											
Is patient able to explain severity of BD symptoms?	9/10	Yes	Yes	Yes	Yes	Yes	Yes	Yes	No	Yes	Yes
Was indication for LT according to clinical guidelines?	8/10	No	Yes	Yes	Yes	Yes	Yes	?	Yes	Yes	Yes
Is patient able to explain why s(he) is/was taking lithium?	8/10	No	Yes	Yes	Yes	Yes	Yes	Yes	No	Yes	Yes
Were side effects of lithium explained?	1/10	No	Yes	No	No	No	No	No	No	No	No
Nephrological follow-up lithium											
Was kidney function checked at least 2x/year?	8/10	Yes	No	Yes	Yes	Yes	Yes	Yes	Yes	No	Yes
Were lithium levels checked every 6 months?	9/10	Yes	Yes	Yes	Yes	Yes	Yes	Yes	Yes	No	Yes

*Psych* psychiatrist, *GP* general practitioner, *neph* nephrologist, *n.a.* not applicable (e.g. because this stage//symptoms went undetected), *?* unknown to patient, *LT* lithium therapy

The patients’ views on LT are distinctly diverse, with four patients reporting negative experiences voicing a fervent wish to have the drug banned or strongly preferring the agent to no longer be prescribed.

*“When I started taking lithium, I developed a whole host of side effects,… but at some point I still agreed to continue when it proved to do a good job”*- *patient 2.*

*“I developed kidney problems, not by my own doing but through the medication for crying out loud, and which I find very hard to deal with”*- *patient 6*

Those with a more moderate view indicated they thought it was a good drug but that it should not be prescribed for uninterrupted long-term use or that another drug should be tested first, with lithium being prescribed only when the former agent proved ineffective.

*“Lithium is good, but not for such a long period”*- *patient 10*.

## Treatment of bipolar disorder

Mean age at BD diagnosis was 40.4 years (range: 30–62 years). Before they were started on lithium, six patients had been hospitalised. In eight of our ten patients a psychiatrist was involved in initiating and monitoring LT, while in the other two this was done by their GPs. Average duration of LT was 25 years (range: 6–43 years), during which period four patients had experienced lithium intoxication. Two patients were unable to explain why they had been or were taking lithium. Eight indicated the drug to work well while one patient could not distinguish its effects from those of a previous drug that had also been effective.

*“If I don’t take lithium I get anxious that I’ll get manic or depressed and I don’t want that”*- patient 9

Three patients were still taking lithium at the time of the interview or had restarted LT, one of whom had not been offered an alternative after LT discontinuation, while in one the new agent was less effective and LT was restarted. The third patient had stayed on lithium for fear of a manic relapse.

*“They decided to stop prescribing lithium when they found out that my kidneys were acting up. But I got terribly depressed.. got into a deep depression, which is why we jointly decided that I should start taking it again*”- patient 3

The remaining seven patients no longer took lithium. One patient experienced a manic episode after LT cessation without receiving replacement therapy. After starting a new regimen, the patient’s mental state stabilised. Five patients were clear in indicating that after LT discontinuation they had experienced no relapses while being on an alternative agent. Two no longer took any psychotropic medication and had remained symptom-free.

## Experiences with kidney failure and RRT

Nine of the interviewees were receiving RRT, with a mean of 2.6 years (range: 1 month to 10 years). None had experienced an increase in bipolar symptoms when first starting haemodialysis. One patient was admitted after 6 months of dialysis on account of a psychotic depression. Despite reporting various RRT-related problems, such as shunt problems and time burden, the majority (7/9) indicated having grown used to RRT and feeling well.

*“I have such a huge fear of blood… and I needed to press the shunt for such a long time! 45* *min or even longer”*- patient 3

“*I could not sit… the pain when they put the needle in* - *leaning back makes you feel really sick”*- patient 8

## Information on lithium and kidney-function monitoring

None of the interviewees remembered having been offered a choice in treatments when starting lithium (Table 1). Accordingly, all viewed the decision to start LT as paternalistic, with a sense of discontent being most pronounced. Only one patient reported having been informed about the side effects of lithium (Table 1).

Kidney function and lithium levels were monitored regularly in seven patients. At the time when serious deterioration of renal functions was established (which stage was identified in six of the ten patients by the attending clinician), again, none of the patients remembered treatment options being discussed. When ESRD was diagnosed, however, patients and family members were more actively involved in treatment decisions.

*“I asked if I could start taking lithium again, and the kidney specialist and psychiatrist jointly decided I could”*- *patient 3*

*“My psychiatrist got in touch with the nephrologist and, with our kids there, we all got to thinking that restarting lithium would be a good thing to do”*- *patient 5*

*“If I would start feeling anxious again, I know I can ask to have me restarted on lithium”*- *patient 7*

## Patient recommendations for clinicians and fellow or future patients

Asked what could be done to improve patient education and information, most of the interviewees (6/10) recommended to provide more information about lithium including its adverse effects. It was further recommended (2/10) to take someone with you to such appointments and that during the consultation the patient should be offered a choice of treatments (2/10). It was additionally suggested that a hand-out might be useful, to allow some time between the visit and the decision, and to possibly follow-up on the discussion every one or 2 years.

*“I didn’t get no information or anything, I wish they’d explained things to me”* - *patient 1*

*“I want to hear what medicine he’s going to give me. And that he says: you have this medicine, and this and another. And this one works like this and the other one like so. And that you can choose”*- *patient 6*

Four patients furthermore hoped that patients first being prescribed lithium will be monitored better. Two patients expressed a wish for more visits and dialogue rather than pills, while new patients are advised to be well aware of the consequences of LT. Finally, the advice to first try another drug treatment was offered frequently.

*“Talk to patients more, don’t just give them a pill right away”*- patient 1

*“You can’t go and drug patients up just like that…you need to communicate”*- patient 8

## Discussion

To our knowledge, ours is the first survey to report on the experiences of ten patients with bipolar disorder and lithium-induced end-stage renal failure. Since the literature on the specific wishes of patients with regard to BD treatments is limited (Fisher et al. 2017), we purposely contacted patients who were likely to be dissatisfied with the results of their treatment to learn from their experiences.

### Patients want to be given options

Although small-scale and explorative, our study did yield clear patient preferences regarding the decision-making process. The majority of the patients we interviewed labelled the information they had received about the treatment of their BD and the treatment decision as paternalistic. Even though at the time they had given their informed consent, they all indicated they would have preferred to have been more involved in the decision to start LT. In addition to more information on lithium, more options should have been discussed in terms of the benefits and disadvantages of different medications, refraining from medication, psychotherapy, or lower doses.

It is plausible to assume that when our patients were first started on LT, open discussions about treatment options was not the commonplace practice it is today. Still, whether in today’s consulting rooms shared decision making is truly shared and on an equal par still is a question of debate (Verwijmeren and Grootens, under review; Alguera-Lara et al. 2017). Patients not always make well-balanced rational decisions; they also base their decisions on the views of their doctors in whom they have put their trust (Fraenkel and McGraw 2007). Another recent BD study found that it primarily are clinician-related factors that determine treatment modalities, whereby patient preferences play a lesser role (Fisher et al. 2017). This practice thus contradicts the wish for shared decision making patients express (Fisher et al. 2017; Alguera-Lara et al. 2017).

### Limitations

Our study has several limitations. It firstly suffers from a selection bias in that for the vast majority of people living with BD lithium is effective and has no nephrogenic effects. Moreover, patient preferences may change in the course of the illness (Hajda et al. 2016). We interviewed patients who had undergone all treatment phases and were asked to retrospectively contemplate what would have been the optimal process, which ideas may have differed from those at the time they first started LT, while recall bias could have affected outcome determination. Some of the events were a long time ago, and it is a well-known fact that patients cannot remember all what is explained and told in the consulting room. Though, in two cases, the spouse participated in the interview and confirmed the answers given by the patient. Furthermore, high emotional levels and psychodynamic defence mechanisms (such as denial) may have contributed to the current descriptions of their medical history and the roles of themselves and their doctor.

Unfortunately, these confounders also prevent us from asking the attending clinicians to consider their treatment decisions in retrospect.

### Recommendations

Kidney dysfunction is not always preventable and sometimes renal replacement therapy is accepted as part of the deal because lithium is indispensable for the patient concerned. The ultimate decision should always be tailored to the individual. In today’s clinical practice the final decision lies with the patient and not with the clinician. It is then crucial that each patient is well informed about ‘lithium and the kidney’ to enable them to make sound decisions as to their treatment. Patients with complex mood disorders often have symptom-free intervals during which they are receptive for detailed, targeted information and instructions while those closely involved in the patient’s life may be invited to also attend such visits. Since the course of renal function loss tends to be insidious, there is no urgent need to acutely cease LT or switch to another agent, allowing all parties sufficient time to make informed decisions and try out different regimens.

In Table 2 we offer recommendations on LT and potential renal effects. We have adopted a nuanced approach in which we have incorporated the patient’s perspective, which is in contrast to the current nephrological guidelines that stipulate to discontinue lithium ‘top down’ with a GFR < 60 in the presence of an intercurrent illness that increases the risk of acute renal failure. We distinguish three stages during LT, with stage 1 comprising the start of the treatment, stage 2 the first signs of mild to moderate reduction in renal functions (GFR 45–59 ml/min/1.73m^2^), and stage 3 the moment severe renal insufficiency or failure is diagnosed. In our recommendations we merely provide cues for what needs to be done and, as yet, not how to do so or which alternatives can or should be considered. We invite all parties to participate in initiatives that will help us enhance the knowledge and decision-making skills regarding lithium for both patients and clinicians.

**Table 2:** Recommendations for health professionals regarding lithium therapy and potential renal complications

Stage 1: Start lithium therapy (GFR > 60 ml/min/1.73 m^2^) Relevant for all patients on lithium	Discuss symptoms/course of mental disorder and provide information on lithium and treatment options on a regular basis Discuss lithium-related complications such as nephrogenic diabetes insipidus and renal failure (esp. in euthymic phase) Discuss lifestyle factors (smoking, body weight) Provide information about lithium use (dose/duration), prevention of intoxication, and how to act in case of dehydration Initiate lithium and kidney monitoring in accordance with (inter)national guidelines Attending health professional(s) should recognise and act on first signs of declining renal function at an early stage (i.e. increasing creatinine levels but also decreasing GFR)
Stage 2: First signs of renal dysfunction (GFR 40-60 ml/min/1.73 m^2^) Relevant for 12% of patients taking lithium	Attending health professional(s) should recognise and act on first signs of declining renal function at an early stage (i.e. increasing creatinine levels but also decreasing GFR) Intensify monitoring Obtain advice from or refer for treatment to experts in the field of lithium-induced nephropathy (preferably a nephrologist and psychiatrist) Explain end-stage renal disorder, prognosis, and implications of haemodialysis Discuss all pros and cons of all relevant treatment options with patient and family (e.g. continuing lithium therapy, tapering lithium, switching to another drug) Take into consideration that renal dysfunction will progress at GFR < 40 (‘point of no return’).(5)
Stage 3: Severe renal insufficiency and renal failure (GFR < 25 ml/min/1.73 m^2^) Relevant for 12‰ to approx. 1% of lithium users	Treatment by psychiatrist and nephrologist If lithium is discontinued: review regularly whether lithium should be restarted.

### Quality of treatment

It needs to be noted that we conducted our survey in the Netherlands, a densely populated and wealthy country sporting numerous lithium outpatient clinics and a national network of knowledge centres in which patients and patient associations actively participate. With our study we have no intention of praising or criticising our national healthcare policies. Our only aim is to give a small group of patients a voice and to learn from them. Nonetheless, in retrospect, treatment decisions and delivery might have been different. Two of the ten LTs were not monitored by a psychiatrist and it was still decided to taper lithium or to switch to another mood stabiliser.

It is of the utmost importance that LT is delivered and monitored by psychiatrists who, in addition to symptom-contingent cues, can also identify and discuss time-contingent complaints with their patient and can keep in close contact with the attending nephrologist. It is in the patients’ best interest that they are seen at a lithium clinic that is equipped to identify the absence of (regular) renal monitoring and detect insidious renal dysfunction in a timely manner, and where staff has expert knowledge about lithium-induced renal damage so that they can be optimally informed about the options available to them. Our survey demonstrates that this is exactly what the patients who have to cope with a serious mood disorder on a daily basis want and expect.

## Conclusions

- This is the first paper that focuses on the opinions of lithium patients who developed end stage renal disease.
- Retrospectively, the treatment and monitoring of lithium and the information process were inadequate in many cases.
- We give suggestions on how to inform patients taking lithium for their BD timely and adequately on the course of renal function loss in the various stages of their treatment.

## Abbreviations

BD: bipolar disorder
CKD: chronic kidney disease
ESRD: end stage renal disease
GFR: glomura
LT: lithium therapy
